# Imaging of non-neuronopathic Gaucher disease: recent advances in quantitative imaging and comprehensive assessment of disease involvement

**DOI:** 10.1186/s13244-019-0743-5

**Published:** 2019-07-10

**Authors:** Andrew J. Degnan, Victor M. Ho-Fung, Rebecca C. Ahrens-Nicklas, Christian A. Barrera, Suraj D. Serai, Dah-Jyuu Wang, Can Ficicioglu

**Affiliations:** 1 Department of Radiology, Children’s Hospital of Philadelphia, 3401 Civic Center Blvd., Philadelphia, PA 19104 USA; 2 Department of Radiology, Perelman School of Medicine at the University of Pennsylvania, 3400 Civic Center Blvd., Philadelphia, PA 19104 USA; 3 Division of Human Genetics, The Children’s Hospital of Philadelphia, Colket Translational Research Building, 3501 Civic Center Blvd, Floor 9, Philadelphia, PA 19104 USA; 4 Department of Pediatrics, Perelman School of Medicine at the University of Pennsylvania, 3400 Civic Center Blvd., Philadelphia, PA 19104 USA

**Keywords:** Gaucher disease, Lysosomal storage disorder, Bone marrow infiltration, Quantitative MRI, Treatment monitoring

## Abstract

Gaucher disease is an inherited metabolic disorder resulting in deficiency of lysosomal enzyme β-glucocerebrosidase causing the accumulation of abnormal macrophages (“Gaucher cells”) within multiple organs, most conspicuously affecting the liver, spleen, and bone marrow. As the most common glycolipid metabolism disorder, it is important for radiologists encountering these patients to be familiar with advances in imaging of organ and bone marrow involvement and understand the role of imaging in clinical decision-making. The recent advent of commercially available, reliable, and reproducible quantitative MRI acquisitions to measure fat fractions prompts revisiting the role of quantitative assessment of bone marrow involvement. This manuscript reviews the diverse imaging manifestations of Gaucher disease and discusses more optimal quantitative approaches to ascertain solid organ and bone marrow involvement with an emphasis on future applications of other quantitative methods including elastography.

## Key points

- Non-neuronopathic Gaucher disease most conspicuously involves bone marrow, liver, and spleen.
- Organ volumes are commonly used for disease severity and treatment response.
- Anatomic imaging is relatively insensitive to marrow infiltration in Gaucher disease.
- Quantitative marrow fat-fractions indicate musculoskeletal severity and show early treatment response.
- Elastography may aid in assessing fibrosis in Gaucher disease.

## Background

### Classification of Gaucher disease

As the most common lysosomal storage disorder, Gaucher disease manifests with abnormal accumulation of glucosylceramide within macrophage lysosomes in multiple organs resulting from an absence or deficiency of β-glucocerebrosidase (also termed β-glucosidase, a glycosphingolipid) enzyme activity [1, 2]. This disease is inherited in an autosomal recessive fashion with over 350 mutations in the glucosylcerebrosidase (*GBA*) gene identified to-date [3].

Although this condition is attributed to a single gene, there is substantial variability in the phenotypic presentation of Gaucher disease with three classically described types [3]. This article focuses on the chronic, non-neuronopathic form (type 1), manifesting with prominent involvement of the reticuloendothelial system with liver, spleen, and marrow involvement, whereas the other types (acute neuronopathic type 2 and subacute-chronic neuronopathic type 3, summarized in Table 1) are far less common [4]. It is worth noting that some experts propose conceptualizing these disease types as a phenotypic spectrum rather than as distinct entities. Some work has suggested subclinical neurological involvement in the “non-neuronopathic” type with abnormal brain magnetic resonance spectroscopy (MRS) findings reported in type 1 Gaucher disease patients, although subsequent investigations have failed to replicate these observations and further work is needed [5, 6]. Others have suggested an increased risk of Parkinsonism in individuals with *GBA* mutations that reflects a complex relationship between glucocerebrosidase and Lewy body disorders [7].

**Table 1:** Clinical classifications of Gaucher disease

Type	Name	Dominant clinical manifestations	Predilections	Age of onset	Life expectancy	Treatment
1	Chronic, non-neuronopathic	Prominent visceral involvement Anemia, bleeding predilection Osseous manifestations (avascular necrosis, fracture) Growth impairment	N370S mutations Ashkenazi Jews	Variable (childhood-early adulthood)	Normal to almost-normal	ERT or SRT for symptomatic patients
2	Acute, neuronopathic	Severe neurological involvement (supranuclear gaze palsy, strabismus, opisthonus) Lung involvement	None	Neonatal-infantile	Poor (neonatal or infantile demise)	Supportive
3	Subacute-chronic, neuronopathic	Progressive neurologic involvement and cognitive deterioration (myoclonic seizures, supranuclear gaze palsy) Variable visceral involvement	L444P, D409H mutations Arab and Japanese populations	Variable (childhood-adulthood)	Shortened, variable (childhood-early/mid-adulthood)	ERT for visceral involvement

While type 1 Gaucher disease is most often diagnosed in childhood or early adulthood with a little under one-third of cases diagnosed by 10 years of age [8], this condition can come to clinical attention at any age due to gradations of residual enzyme activity and phenotypic variability [9]. Pediatric phenotypic expression, however, tends to be particularly severe [10]. The diversity of clinical manifestation and spectrum of disease severity underscore the importance of accurate assessment of multisystemic involvement at diagnosis and across the lifespan [9]; as this article will further discuss, diagnostic imaging plays an instrumental role in clinical evaluation and management of Gaucher disease.

### Epidemiology, clinical evaluation, and treatment

While Gaucher disease is relatively uncommon overall with an estimated prevalence of approximately 1/40,000, certain groups have much higher carrier and disease prevalence with a carrier frequency of as high as 6–10% in Ashkenazi Jewish individuals [11, 12]. The majority of type 1 Gaucher disease patients are diagnosed during childhood, and type 1 Gaucher disease is most commonly due to biallelic p.*N370S* mutations in the Western world [4].

Detailed description of the diagnostic workup and clinical management of Gaucher disease is beyond the scope of this article, but it is helpful for radiologists evaluating patients with Gaucher disease to be familiar with commonly utilized clinical tests and available therapies. Laboratory tests and biomarkers are summarized in Table 2; these clinical tests are part of comprehensive patient assessment that also involves diagnostic imaging. Clinical severity scoring is often performed using the Gaucher Disease Type 1 Severity Scoring System (GD-DS3) [13], which incorporates bone involvement, hematologic parameters, and organ enlargement. The Pediatric Gaucher Severity Scoring System (PGS3) [14] additionally accounts for growth disturbance as a key consideration in the younger population.

**Table 2:** Laboratory investigations in Gaucher disease

Laboratory investigation	Basis	Advantages	Disadvantages
Angiotensin-converting enzyme	Increased in the plasma of affected patients	Decreases with treatment	Nonspecific
Beta-glucocerebrosidase activity assay	Direct assessment of enzyme responsible for disease	Gold standard test Elevated in active disease	Expense
Bone marrow aspirate	Visualization of Gaucher cells in marrow	Identification of alternative or concomitant disease entities with similar presentations (e.g., hematologic malignancy)	Not routinely recommended if Gaucher diagnosis is highly suspected Nonspecific (pseudo-Gaucher cells) Discomfort Expense
CCL18	Produced by Gaucher cells as macrophage chemokine	Elevated in active disease Suitable in chitotriosidase deficient individuals More closely reflects organ volumes than chitotriosidase	Expense No head-to-head comparison with chitotriosidase
Chitotriosidase	Released by glucocerebrosidase-laden Gaucher cells	Elevated in active disease Reduction from baseline values indicates treatment response Increasing values are consistent with active disease	Normal individuals occasionally may not produce chitotriosidase Can vary widely between patients Expense
DNA sequencing	Testing for genetic mutations (known and de novo) in the *GBA* gene	Provides detailed information regarding genotype, which may be associated with specific forms of the disease Identifies carriers	Expense Variable phenotypic expression
Ferritin, serum iron, iron binding capacity	Iron overload occurs in patients. Uncertain etiology with possible association with *HFE* gene mutations, chronic inflammation	Correlates with hepatomegaly Decreases with treatment	Nonspecific with poor correlation with organ iron deposition on imaging and disease severity scoring
Glucosylsphingosine	Byproduct related to glucosylceramide, reflecting beta-glucocerebrosidase function	Correlates with other markers of disease activity, organomegaly, platelet levels Decreases with treatment	Expense, availability
Liver function tests (AST, ALT, bilirubin, albumin, total protein)	Hepatic dysfunction related to liver infiltration is common	Provides assessment of active hepatic involvement	May be insensitive to early hepatic involvement
Routine hematological tests (hemoglobin, platelet count, coagulation parameters)	Anemia and thrombocytopenia hallmark features of this disease	Provides information regarding hematologic involvement that may prompt other treatment	Nonspecific for overall disease severity
Tartrate-resistant acid phosphatase	Marker of osteoclasts and Gaucher cells	Decreases with treatment	Nonspecific

Traditionally, Gaucher disease has been conceptualized as a single gene-related disease due to one enzyme with one treatment consisting of enzyme replacement therapy (ERT) [3]. Prior to the advent of ERT, treatment was largely based on addressing symptoms with splenectomy performed for extensive splenic infiltration in severe patients with poor prognosis. The earliest effective ERT, alglucerase, was introduced in the 1990s and has dramatically changed the prognosis of this condition. Subsequent newer forms of ERT with improved manufacturing techniques and safety profiles have been approved. Newer oral substrate reduction therapy (SRT) functions earlier in the biochemical pathway by mitigating the accumulation of glucosylceramide [15]. Both types of therapies are expensive, and each have disadvantages to consider with ERT requiring intravenous infusion every few weeks and SRT possessing a narrower safety profile with CYP2D6 and CYP3A metabolism considerations as well as being only approved for adult use [3, 4, 16]. Historic and currently available treatment options are summarized in Table 3, but we refer interested readers elsewhere for detailed description of clinical trials and advances in Gaucher disease treatment [17].

**Table 3:** Historic and available therapies for Gaucher disease

Medication name	Therapy type	Advantages	Disadvantages
Alglucerase (Ceredase, Genzyme corporation)	Enzyme replacement	Earliest therapy with demonstrated improvements in organ involvement, biomarkers, bone pain. Satisfactory safety profile	No longer available Derived from human placenta Intravenous route Cost Does not cross blood-brain barrier Allergic reactions
Imiglucerase (Cerezyme, Genzyme corporation)	Enzyme replacement	Replaced alglucerase with comparable therapeutic response Satisfactory safety profile	Intravenous route Cost Does not cross blood-brain barrier Allergic reactions
Velaglucerase alfa (VPRIV, Shire Human Genetics Therapies)	Enzyme replacement	Fewer allergic reactions Comparable therapeutic response with imiglucerase	Intravenous route Cost Does not cross blood-brain barrier
Taliglucerase alfa (Elelyso, Pfizer Inc.)	Enzyme replacement	Easier manufacturing, lower cost	Intravenous route Does not cross blood-brain barrier Less therapeutic response data
Miglustat (Zavesca, Actelion)	Substrate reduction	Oral route Potential to cross blood-brain barrier	Failed to achieve neurological treatment response High prevalence of side effects Cost
Eliglustat (Genzyme corporation)	Substrate reduction	Oral route Early clinical evidence of treatment response	Drug-drug interactions CYP2D6 and CYP3A metabolism considerations Cardiotoxicity Does not cross blood-brain barrier Cost

Many asymptomatic patients with mild disease may not receive treatment at first; most children with milder genotypes perhaps may not require treatment initially. However, active bone disease, even if asymptomatic, and significant hepatosplenomegaly are considered indications for initiating ERT in asymptomatic children [18]. Therefore, monitoring of patients not currently on therapy with imaging is clearly important in guiding clinicians as to early disease involvement [18].

As some patients may respond poorly to particular therapies, imaging can be important to inform clinicians regarding changes in an individual patient’s response to specific therapies to provide personalized therapy decisions. The management of treated individuals who are asymptomatic despite biomarker and imaging findings of Gaucher disease poses a unique challenge, leading some clinicians to propose a concept of minimal disease activity wherein some treated patients may have mild persistent abnormalities on imaging and laboratory investigations without clinically significant disease [19]. Moreover, the substantial financial burden incurred by these therapies with annual treatment costs ranging between 70,000 and 380,000 USD [8, 20] may also motivate the use of imaging assessment of treatment response in well-controlled patients to assist in verifying efficacy and safety of reduced dosing schedules or alternative strategies to reduce the overall cost of therapy.

## Imaging of Gaucher disease involvement

Due to the reliance on ERT and SRT for the long-term management of patients with Gaucher disease, non-invasive imaging methods are essential in ascertainment of response to therapy and detection of disease-related complications. Therefore, understanding the role of different imaging modalities in the context of disease processes is important to provide clinicians and radiologists with guidance in assessing disease severity and treatment response. General strengths and limitations of each imaging modality are summarized in Table 4 [18, 21–24].

**Table 4:** Imaging modalities relevant to Gaucher disease

Imaging modality	Gaucher disease manifestations	Advantages	Limitations
Magnetic resonance imaging without intravenous contrast	Abdominal: organ enlargement, heterogeneous parenchymal signal, hepatic and splenic lesions, decreased ADC in affected organs Musculoskeletal: abnormal marrow signal, avascular necrosis, fracture, vertebral height loss	Offers both qualitative and quantitative multisystem assessment including treatment response Non-invasive, no ionizing radiation Reproducible	Expense Contraindications in selected individuals Sedation requirement for certain patients
Quantitative chemical-shift imaging (QCSI), lumbar spine and proximal femurs	Abdominal and skeletal: decreased fat-fractions	Accurate, reproducible Validated in several studies to correlate with biomarkers and respond to treatment	Availability, technical expertise Expense Cannot be measured in areas of osteonecrosis or vertebral collapse
Magnetic resonance spectroscopy	Abdominal and skeletal: decreased fat-fractions	Accurate, reproducible More reliable at lower fat-fractions than chemical-shift imaging	Limited validation Availability, technical expertise Expense Acquisition time
Magnetic resonance elastography	Abdominal: increased liver and spleen stiffness values	Non-invasive, no ionizing radiation May be obtained in conjunction with other MRI evaluations	Availability Expense Less well-validated
Magnetic resonance imaging with hepatocyte-specific intravenous contrast	Abdominal: organ enlargement, heterogeneous parenchymal signal, hepatic and splenic lesions	Gold-standard for liver lesion characterization Non-invasive, no ionizing radiation Reproducible	Expense Contrast administration-related issues Low-yield in the absence of previously identified suspicious or indeterminate lesion May not avoid need for confirmatory biopsy in Gaucher disease to overlap between liver involvement and suspicious imaging features
DXA	Osteopenia consistent with worsening marrow infiltration	Osteopenia indicates worsening skeletal involvement and may predict pathologic fracture risk Nominal ionizing radiation exposure	Unreliable in sites of osteonecrosis and compression deformity Normative values unreliable below 6 years of age Low-yield in younger children at lower risk of fracture Predictive value of BMD to predict fracture risk in children is undefined
Computed tomography with intravenous contrast	Abdominal: organ enlargement, heterogeneous parenchymal attenuation, hepatic and splenic lesions	Availability Satisfactory identification of lesions	Incomplete characterization of focal lesions Ionizing radiation exposure (may be optimized for dose reduction)
Ultrasound, abdomen	Abdominal: organ enlargement, heterogeneous hepatic echotexture, hepatic and splenic lesions	Accessible, affordable Non-invasive, no ionizing radiation equivalent to CT for screening for liver complications	Operator dependent, protocols sometimes rely on single operator Overlap of benign and malignant focal lesion characteristics requiring additional workup Less sensitive than MRI for comprehensive assessment of organ involvement Disagreement with volumes obtained on other modalities
Chest CT	Interstitial and bronchial wall thickening, groundglass and centrilobular nodular opacities	Accurately depicts pulmonary involvement in patients with symptoms	Pulmonary involvement is rare Findings often nonspecific Ionizing radiation exposure
99 m-Tc-Sestamibi scintigraphy	Increased uptake at distal femoral and proximal tibial epiphyses	Semi-quantitative method May correspond with treatment response Availability	Ionizing radiation exposure Poor spatial resolution Limited validation data Low specificity
99 m-Tc-MDP scintigraphy	Decreased uptake at sites of bone crises	May potentially differentiate between bone crises and osteomyelitis	Not well-validated Ionizing radiation exposure Availability Expense Poor spatial resolution Not specific
Echocardiography and cardiac MRI	Pulmonary hypertension (mostly adults on treatment) Valvular calcifications (D409H homozygous mutation)	Non-invasive, no ionizing radiation Definitive investigations for cardiac involvement	Low-yield in pediatric population Expense Limited data to support widespread use, particularly for cardiac MRI
Acoustic radiation force impulse/shear wave elastography (US)	Increased liver and spleen stiffness values	Similar or higher performance compared with transient elastography Non-invasive, no ionizing radiation May be combined with conventional US evaluation of organ involvement No sedation	Availability Measurement variability
Transient elastography	Increased liver and spleen stiffness values	Non-invasive, no ionizing radiation Expense	No imaging guidance to assess most affected regions of organs No imaging component for further characterization of organ parenchyma quality or lesions

### Hepatic and splenic involvement

#### Organomegaly and infiltration

While the inciting pathophysiological mechanism of Gaucher disease, enzymatic deficiency with accumulation of glucocerebroside, has been elucidated, the extent to which substrate accumulation accounts for liver enlargement has been called into question [25]. Originally, visceromegaly was attributed to simple accumulation glucocerebroside in the reticuloendothelial system with buildup of this material within Gaucher cells in the spleen as well as Kupffer cells in the liver [25]. However, newer evidence suggests that pro-inflammatory and pro-infiltrative chemotaxis factors may also account for organ enlargement with Gaucher cells contributing only partially to enlarged liver and spleen volumes [25, 26]. Regardless, severe hepatic involvement generally corresponds with additional organ system manifestations. Hepatosplenomegaly is a hallmark of Gaucher disease and uniformly present beginning in childhood, and liver disease is a major contributor to Gaucher disease-related mortality [27]. As such, traditional imaging of disease severity has been based on hepatic and splenic visceral organ enlargement [24, 25, 28].

Organ sizes are interpreted from a clinical standpoint in the context of volume relative to the expected volume based on body weight, expressed as multiples of normal (MN) [18]. While there are a variety of available calculations for normal liver and spleen volumes in the imaging literature incorporating age, sex, and body mass index, most Gaucher disease studies utilize a simple weight-based formula for these normative values (normal liver volume (mL) = 25 mL/kg × weight (kg); normal spleen volume (mL) = 2 mL/kg × weight (kg)) that should be consistently employed to avoid confusion across studies [29]. Treatment objectives often include specific targets such as liver volumes of less than 1–1.5 MN and spleen volumes of < 2–8 MN [8, 30].

**Fig. 1:**
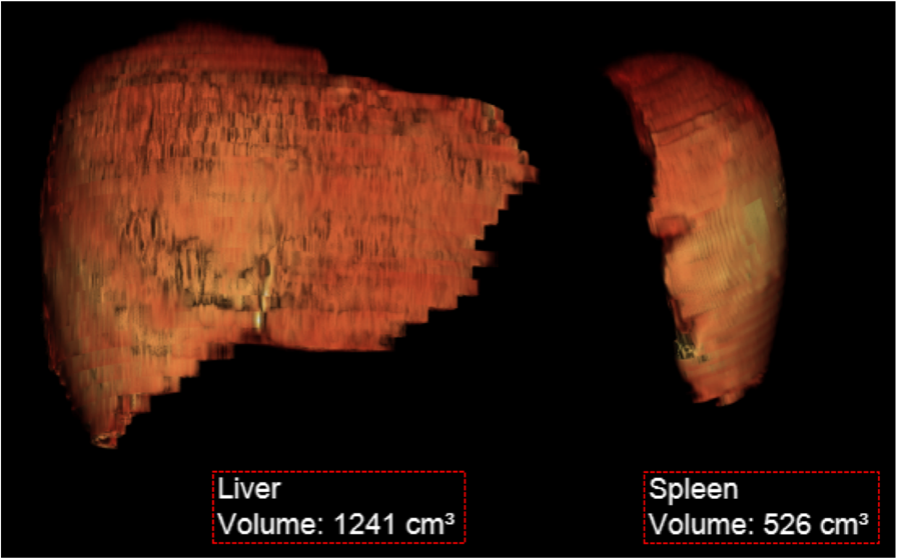
Three-dimensional reconstructions of liver and spleen volumes from MRI in Gaucher disease. Volume-rendered organ volume reconstructions generated from non-contrast MRI data were used to track treatment response in a 9-year-old male with Gaucher disease. Organ volumes for the liver (1241 mL, 1.03 MN) and spleen (526 mL, 5.44 MN) are decreased from a prior examination at 6 years of age (1.35 MN and 6.43 MN, respectively)

Organ volumes may be calculated using a variety of methodologies with the simplest being a volumetric estimate from orthogonal dimensions on conventional ultrasound. Conventional ultrasound has the benefit of being non-invasive and relatively inexpensive; most notably, ultrasound may avoid the use of sedation in younger children [31, 32]. While the longitudinal axis measurements on US modestly correlate with CT-derived volumes, others note that sonographic-derived values differ significantly from true volumetric measurements (Fig. 1) obtained on cross-sectional imaging, namely CT and MRI, and ultrasound may also depend on a single reader over serial evaluations for consistency [31, 33, 34]. Therefore, many consensus recommendations caution against the use of ultrasound [18]. In more recent years, there is widespread agreement upholding the use volumetric MRI over CT, given concerns regarding ionizing radiation exposure from repeated examinations, and additional information obtainable with MRI [24]. Semi-automated techniques of measuring organ volumes on MRI also afford more reliable measurements and avoid dependence on specialized readers [35].

Organ volumes decrease substantially with therapy initiation, although some have noted that well-controlled disease may result in plateauing of organ volumes after the first 3 years of successful treatment [34].

Diffusion-weighted imaging (DWI) offers another quantitative assessment of organ infiltration as a surrogate of tissue cellularity [36]. Apparent diffusion coefficient (ADC) values in the liver and spleen appear to correlate well with chitotriosidase as a marker of disease severity in one pediatric study with decreased ADC connoting greater infiltration and worse involvement [37].

#### Liver fibrosis

While cirrhosis and portal hypertension have been traditionally regarded as rare in Gaucher disease [38], there is increased risk for liver fibrosis, cirrhosis, and hepatocellular carcinoma (HCC) in this condition, especially in previously splenectomized individuals [39–41]. Gaucher cell deposition may establish a fibrogenic microenvironment due to chronic low-grade inflammation [25, 27]. In Gaucher disease, liver fibrosis is thought to increase the increased risk of hepatic cirrhosis, HCC risk, and liver disease-related mortality.

**Fig. 2:**
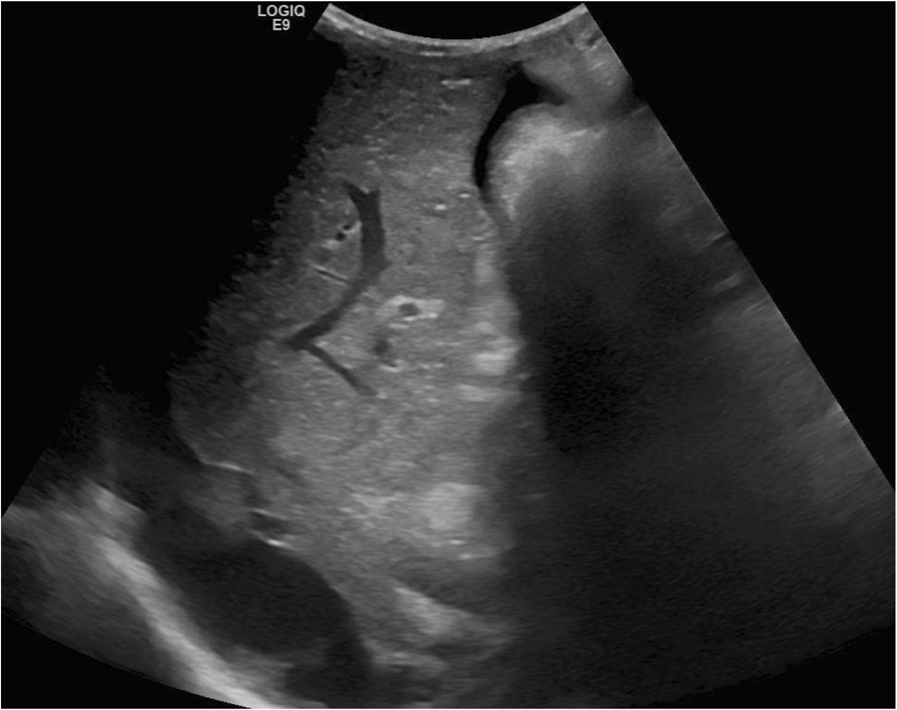
Liver cirrhosis in pediatric Gaucher disease. Sagittal grayscale ultrasound demonstrates an enlarged liver with nodular cirrhotic morphology and perihepatic ascites in a 12-year-old male with Gaucher disease

Advances in the understanding of Gaucher disease and observations of elevated ferritin levels and increased risk of hepatic fibrosis suggest greater importance of imaging assessment for complications of liver involvement beyond simple organ enlargement. Ultrasound has been traditionally used to monitor development of cirrhotic morphology (Fig. 2) but is relatively insensitive in detecting earlier fibrotic changes. Some means of non-invasively assessing hepatic fibrosis include non-imaging-based transient elastography (TE), ultrasound shear wave elastography (SWE), and magnetic resonance elastography (MRE) [42]. These elastography modalities provide reliable measurements that can detect significant liver fibrosis each with unique strengths and limitations summarized in Table 4 [42].

Liver stiffness may be only mildly elevated in Gaucher disease patients without cirrhosis, likely reflecting treatment-related reductions in the extent of liver fibrosis [43]. However, Gaucher disease patients with cirrhosis exhibit markedly elevated liver stiffness values [43], and other studies of patients who were previously splenectomized (with more severe disease) demonstrated greater liver stiffness compared with patients with milder disease [40, 44]. In a recent study, MRE-measured liver stiffness values correlated with Gaucher clinical severity (GD-DS3) scores, highlighting the potential clinical utility of evaluating liver stiffness in this disease [44]. These findings suggest a role for elastography-based modalities in identifying early hepatic fibrosis in Gaucher disease patients, although further study is needed to better understand the extent of fibrosis in Gaucher disease and the clinical utility of earlier identification.

#### Iron overload

Other important clinical manifestations of Gaucher disease include hematologic abnormalities such as anemia and hyperferritinemia. The precise etiology of hyperferritinemia in Gaucher disease is poorly understood, although it is a frequent finding that may correspond with other indicators of disease severity and respond to ERT [45, 46]. Ferritin levels are also noted to be higher in asplenic patients, a finding that may be confounded by greater disease severity in these patients also often requiring blood transfusions [45]. From an imaging standpoint, hyperferritinemia with iron deposition such as within the liver may appear on with increased attenuation on CT, signal drop-out on out-of-phase gradient T1-weighted MRI and low signal on T2*-weighted MRI [47]. A group of investigators highlighted substantial iron deposition (as indicated by R2* values) in treated Gaucher disease patients compared with controls [48]. However, the precise relationship between hyperferritinemia and visceral iron deposition is poorly understood in Gaucher disease and the subject of continued investigation.

#### Liver lesions

The majority of liver lesions encountered in Gaucher disease are thought to be related to focal deposition of glucocerebroside within the Kupffer cells [39]; however, Gaucher disease confers substantial increased risk of malignancy and HCC [49, 50].

**Fig. 3:**
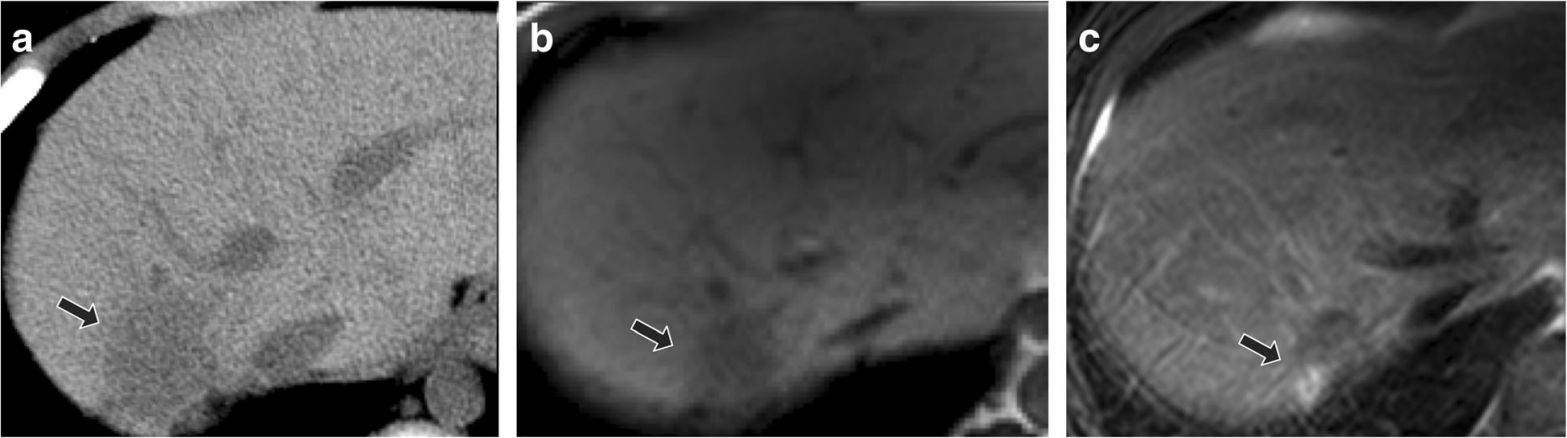
Focal Gaucher cell accumulation in the liver. Axial non-contrast abdominal CT (**a**) demonstrates a focal hypoattenuating hepatic lesion (arrow) in the posterior right hepatic lobe in 10-year-old male with type 1 Gaucher disease. At follow-up at 16 years of age with unenhanced MRI, lesion (arrow) was unchanged in size with T1-weighted hypointense (**b**) and mixed T2-weighted signal intensity (**c**). Findings are most consistent with focal Gaucher cell deposition (“Gaucheroma”)

In one large ultrasound study of Gaucher disease patients, focal liver lesions were identified in just under 5% of individuals; the majority of which were multiple hyperechoic lesions [39]. Authors have suggested that such small, hyperechoic lesions do not merit biopsy if slowly growing, as they are thought to reflect focal accumulation of Gaucher cells [39]. On the other hand, early studies of MRI in Gaucher disease identified focal signal abnormalities in about one-fifth of patients [51]. Such focal accumulations of Gaucher cells are thought to be hypoattenuating on CT, hypointense on T1-weighted imaging, and heterogeneous on T2-weighted imaging (Fig. 3).

In addition, reports of biopsy-proven hepatic amyloidosis have been made in rare numbers of patients [39, 52, 53]. More worrisome, HCC can develop at younger ages in Gaucher disease; the previously mentioned large series identified only one case of HCC, which was found in the setting of rapidly progressive cirrhotic morphology on serial US evaluations [39].

Therefore, screening evaluation for development of suspicious liver lesions is warranted in Gaucher disease. Identification of more suspicious (e.g., hypoechoic, large, irregular, hypervascular) lesions may warrant further investigation with dedicated liver protocol CT, hepatocyte-specific contrast-enhanced MRI, or contrast-enhanced US, which may offer a viable alternative especially for children [54].

#### Spleen fibrosis

**Fig. 4:**
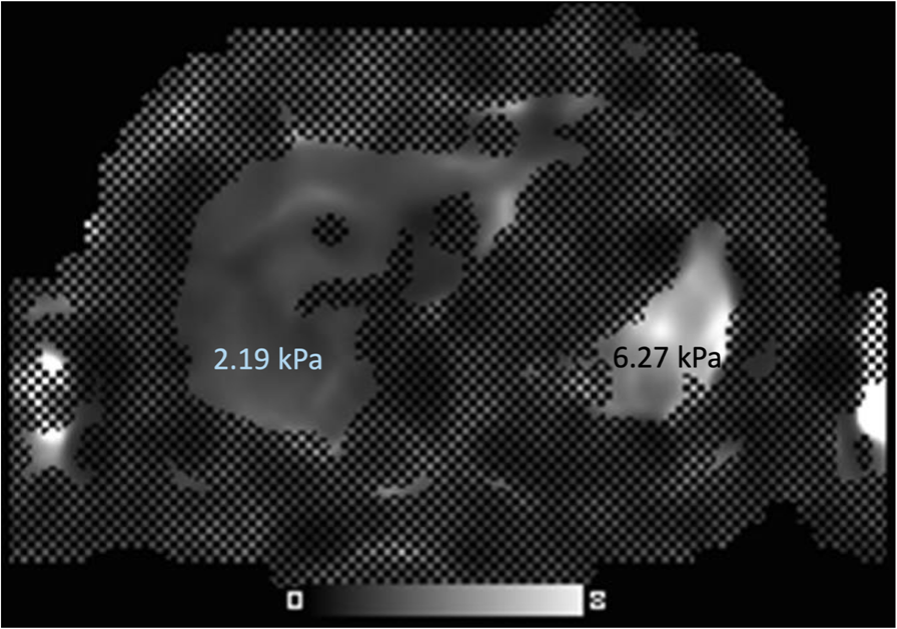
Spleen fibrosis in pediatric Gaucher disease. Axial MR elastography of a 13-year-old patient with newly diagnosed, untreated type 1 Gaucher disease demonstrates elevated spleen stiffness values (6.27 kPa, abnormal defined greater than 3.6 kPa), but no significant hepatic fibrosis identified (2.19 kPa, abnormal defined greater than 2.9 kPa)

Gaucher patients also demonstrate significantly elevated spleen stiffness values using a variety of elastography techniques [43]. Elevated stiffness values observed in Gaucher disease (Fig. 4) may reflect progressive infiltration and inflammatory changes culminating in fibrosis. Elevated spleen stiffness has been deemed a harbinger of portal hypertension in non-Gaucher disease conditions [42]; however, prospective assessment of the clinical significance of splenic fibrosis in Gaucher disease is needed.

#### Splenic lesions

**Fig. 5:**
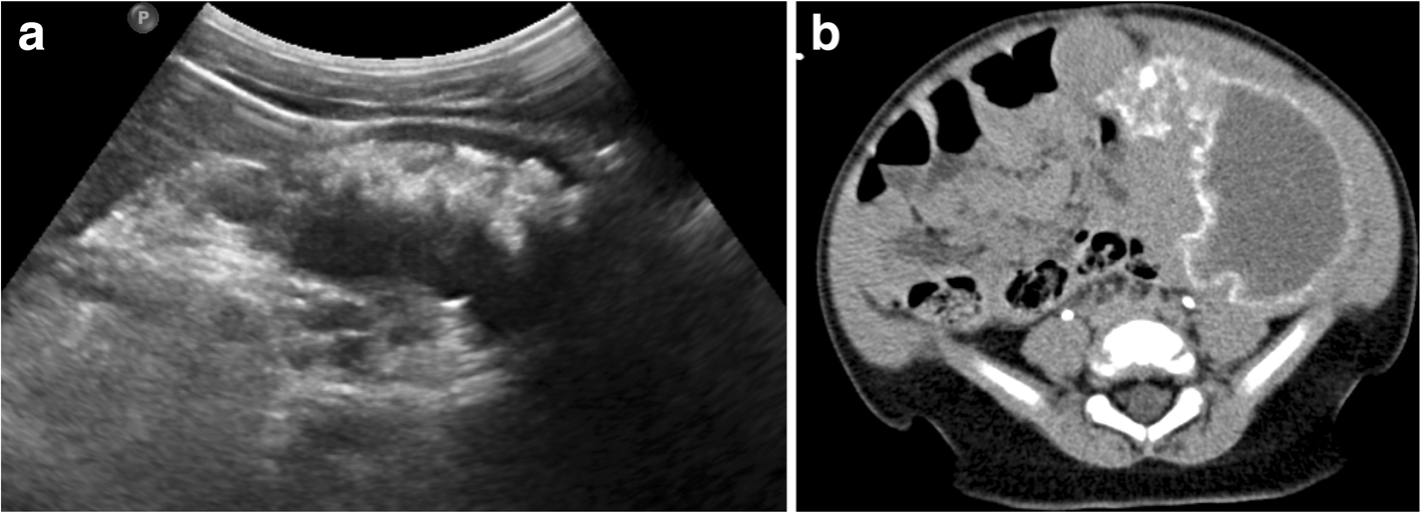
Splenic necrosis in pediatric Gaucher disease. Transverse grayscale abdominal ultrasound (**a**) of a 2-year-old male Gaucher disease patient with marked splenomegaly shows replacement of normal splenic parenchyma with fluid and hyperechoic regions corresponding to dystrophic calcification. Contrast-enhanced abdominal CT (**b**) of this patient demonstrates enlarged spleen replaced with liquefying necrosis and peripheral dystrophic calcifications

Similar to the previously described hepatic lesions thought to represent focal Gaucher cell buildup, patients with Gaucher disease are often noted to have hyperechoic splenic lesions on ultrasound in one-fifth to one-third of cases [43]. These lesions tend to be low in attenuation with occasional peripheral calcification on CT [55]. On MRI, Gaucher-related splenic nodules usually demonstrate low T1-weighted and high T2-weighted signal intensity, although focal areas of necrosis are also encountered which can be hypointense on T1-weighted imaging [32, 55]. Early MRI studies demonstrated that numbers of splenic nodules correlated with spleen size; these investigators posited that these lesions often represent areas of infarction in the setting of splenic infiltration [56]. These splenic nodules appear to be more common in adults, likely reflecting longstanding infiltration needed to result in ischemia [51]. Similarly, subcapsular splenic infarcts were commonly noted as well prior to introduction of effective ERT [51]. In especially severe cases, splenic necrosis may occur with replacement with fluid attenuation material and peripheral calcification (Fig. 5).

### Skeletal involvement and complications

**Fig. 6:**
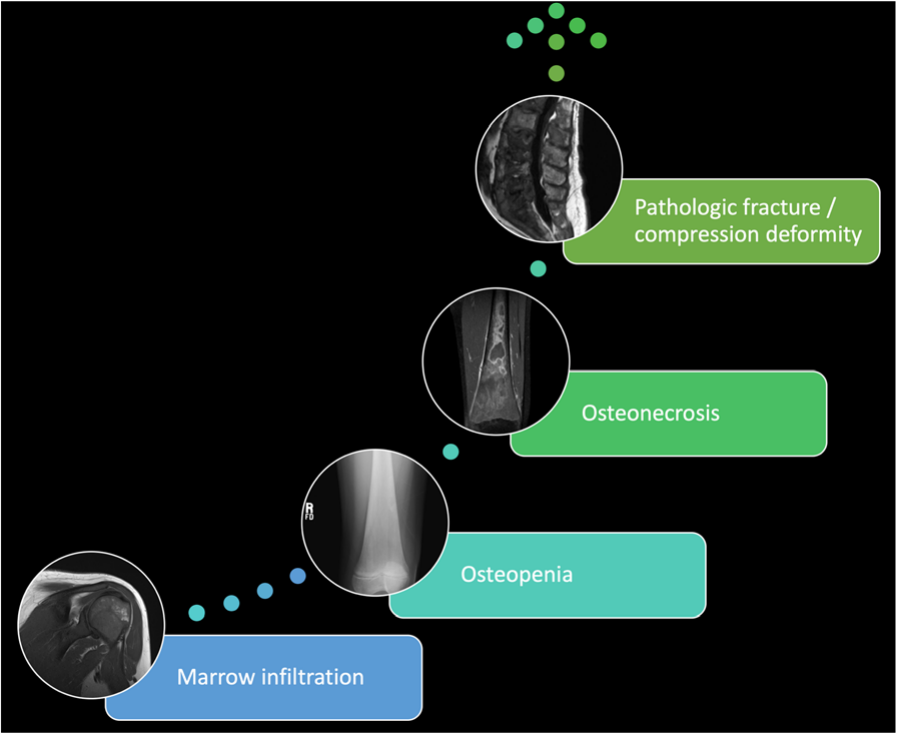
Spectrum of musculoskeletal involvement in Gaucher disease. Nearly all type 1 Gaucher disease patients experience musculoskeletal manifestations ranging from marrow infiltration and osteopenia to pathologic fractures and compression deformities of the spine

In patients with type 1 Gaucher disease, bone marrow involvement represents a spectrum of disease (Fig. 6) with some level of involvement in nearly every patient [4, 57, 58]. Recognition that musculoskeletal manifestations may be the presenting symptom of Gaucher disease is important to avoid confusion with other conditions with overlapping symptoms and signs such as hematopoietic malignancies and rheumatologic conditions [59]. Infiltration of the bone marrow with Gaucher cells with concomitant osteopenia progressively compromises the bone density of the axial then appendicular skeleton with prominent lower extremity involvement of the proximal femurs and tibias [22, 60, 61]. Upper extremity involvement, while less commonly recognized, is also a substantial contributor to musculoskeletal morbidity with many children and adolescents reporting upper extremity pain.

Greater severity of musculoskeletal involvement often corresponds with severe organ involvement. Early investigations of bone involvement in Gaucher disease believed bone changes were irreversible [56]. However, newer studies have shown that treatment results in substantial improvement of early bone marrow changes in many patients. The prognosis of skeletal involvement once complications have occurred remains poor and bone marrow responses take longer than organ responses [55, 62]. Bone turnover biomarkers have largely failed to risk stratify patients, making imaging evaluation of musculoskeletal involvement indispensable to clinical management [57].

#### Marrow infiltration

Abnormal bone marrow involvement in Gaucher disease is a prominent finding, almost uniformly noted in type 1 patients and most conspicuously involves the spine, femurs, tibias, and humeri. Anemia commonly encountered in this condition results in greater hematopoietic marrow than expected for age, and direct accumulation of Gaucher cells also contributes to abnormal bone marrow. Marrow involvement follows a pattern of centrifugal spread with the epiphyses and apophyses mostly spared except in the most severe of marrow infiltration [61]. Bone marrow involvement has long been regarded as more difficult to treat than visceral involvement. A previous study showed through histological examinations a decreased uptake of administered alglucerase within the bone marrow [63]. Marrow infiltration of the lumbar spine is thought to be more responsive to therapy, whereas some authors regard femoral marrow reflecting more static disease [64].

##### Subjective imaging assessment

**Fig. 7:**
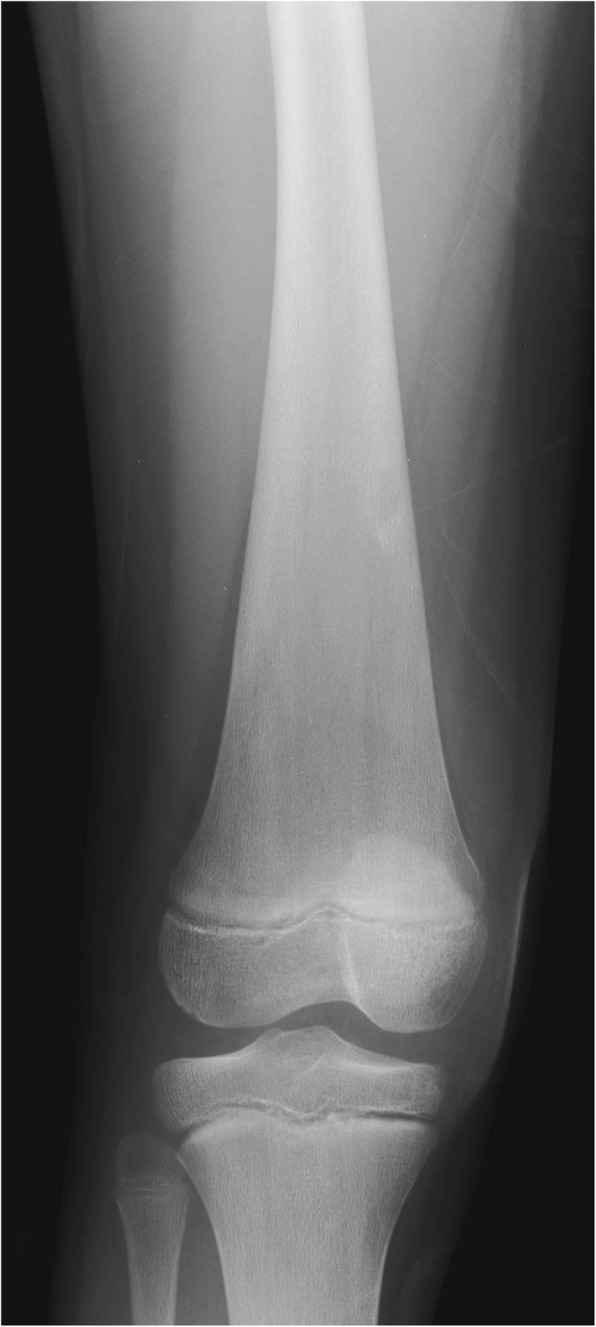
Erlenmeyer flask deformity. Frontal radiograph of the right knee in a 12-year-old-male Gaucher disease patient with widening of the distal femoral diaphysis and metaphysis resulting in Erlenmeyer flask deformity

Radiographs are insensitive for detection of marrow infiltration; substantial medullary cavity expansion is needed to result in the classically reported Erlenmeyer flask deformity of the distal femur (Fig. 7) [22, 65]. In addition, the Erlenmeyer flask finding is nonspecific for Gaucher disease, being encountered in a variety of other conditions reviewed at-length elsewhere [66]. Initial marrow infiltration can be patchy, also making radiographic detection impossible [67].

**Fig. 8:**
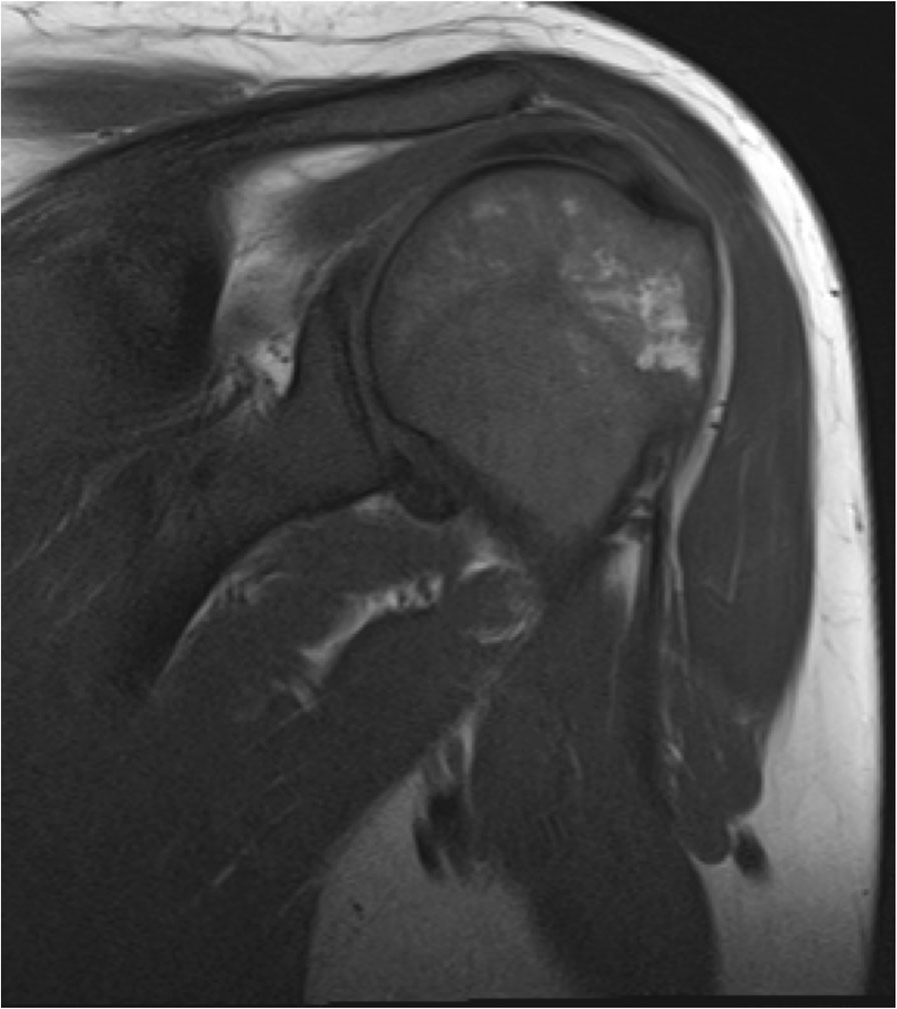
Marrow infiltration in Gaucher disease. A 30-year-old female with type 1 Gaucher disease with shoulder pain. Coronal T1-weighted imaging demonstrates diffuse hypointense infiltration of the humerus including the proximal epiphysis compared to subcutaneous fat

Marrow infiltration manifests with low signal on T1-weighted imaging (Fig. 8). Early qualitative MRI assessments of marrow involvement in patients on ERT-based noted T1-weighted signal increases corresponded with reduced organ volumes with changes observed as early as 1 year [46, 62]. This increase in T1-weighted signal is thought to reflect greater accumulation of yellow marrow with decreased concentration of Gaucher cells and hematopoietic marrow. Interpretation of qualitative marrow changes in children should be interpreted within the context of developmental changes in hematopoietic marrow [10, 68].

##### Semi-quantitative imaging assessment

Numerous semi-quantitative scoring methods have been developed to report overall extent of bone marrow involvement based on signal intensity alterations on MRI, each with specific objectives and limitations summarized in Table 5 [61, 69]. Semi-quantitative region-of-interest (ROI)-based techniques have been offered up as slightly more objective alternatives to signal intensity based methods; the most commonly performed of these methods is the vertebra disc ratio (VDR) in which ROIs are placed on T1-weighted images of the lumbar spine [70, 71]. Out of these methods, the bone marrow burden (BMB) score (Table 6) based on conventional lumbar spine and femur MRI is the most validated and correlates well with disease severity, splenectomy status (indicative of more severe disease), treatment response, and quantitative fat fractions [20, 23, 64, 72–75]. Another method, the Spanish-MRI (S-MRI) score, also incorporates imaging of the pelvis [76]. More recent investigations have applied some of these semi-quantitative scoring techniques to whole-body MRI to assess treatment response [77–79]. While the reliability of these measures is promoted by several small studies, more recent work has called reliability of BMB scoring into question with poor interobserver agreement and others have pointed out issues related to later fat marrow conversion in children [77, 80].

**Table 5:** Semi-quantitative Gaucher disease bone marrow involvement scoring systems

Classification	Bone marrow burden (BMB)	Spanish MRI (S-MRI)	Terk	Rosenthal staging	Duseldorf bone marrow disease score	Vertebra-disc ratio
Sites	Femurs Lumbar spine	Femurs Lumbar spine Pelvis	Femurs	Lower extremities	Lower extremities	Lumbar spine
Considerations	Most validated method Correlates with quantitative Modified method allows use of STIR acquisition Confusion with red marrow in younger patients	Less validated Relevance of pelvis imaging is questioned Confusion with red marrow in younger patients	Does not appreciate more reversible changes seen within the axial skeleton Confusion with red marrow in younger patients	Less sensitive than methods including lumbar involvement Confusion with red marrow in younger patients	Correlates with severe disease Less sensitive than methods including lumbar spine	Quantitative region-of-interest based measurement Limited clinical validation data

**Table 6:** Bone marrow burden (BMB) classification

	Femurs		Lumbar spine	
Involvement	Site	Score	Site	Score
	Diaphysis	1	Patchy	1
	Proximal epiphysis/apophysis	2	Diffuse	2
	Distal epiphysis	3	Absence of fat in basiverteral region	1
Sequence	Signal intensity^a^	Score	Signal intensity^b^	Score
T_2_-weighted, (or, STIR)^c^	Hyperintense	2 (2)	Hyperintense	2 (2)
	Slightly hyperintense	1 (1)	Slightly hyperintense	1 (1)
	Isointense	0 (0)	Isointense	0 (0)
	Slightly hypointense	1 (N/A)	Slightly hypointense	1 (N/A)
	Hypointense	2 (N/A)	Hypointense	2 (N/A)
	Mixed type	3 (3)		
T_1_-weighted	Slightly hyperintense or isointense	0	Slightly hyperintense	0
	Slightly hypointense	1	Isointense	1
	Hypointense	2	Slightly hypointense	2
			Hypointense	3
		Sum		Sum

^a^Relative to subcutaneous fat

^b^Relative to normal intervertebral disc

^c^Modified BMB scoring with STIR instead of T_2_-weighted imaging, adjusted scoring indicated in parentheses

##### Quantitative imaging assessment

Multiple quantitative factors have been proposed to objectively assess marrow involvement in Gaucher disease and are supported by histopathologic evidence of an inverse relationship between marrow glucocerebroside and triglyceride concentrations [72]. Some of earliest quantitative assessments of bone marrow composition were performed using dual-energy CT and noted low amounts of marrow fat in patients [81, 82]. These initial investigations envisioned fat fractions as a marker of bone marrow disease severity with lower values representing greater infiltration [81]. Early MRI investigations noted longer T1 relaxation times in the bone marrow as well as decreases in these values with treatment [83, 84]. The emergence of MRI techniques shortly following these CT investigations led to the abandonment of CT for this indication, and fat fractions calculated from MRI have become the dominant quantitative variable to report marrow involvement [61, 84].

**Fig. 9:**
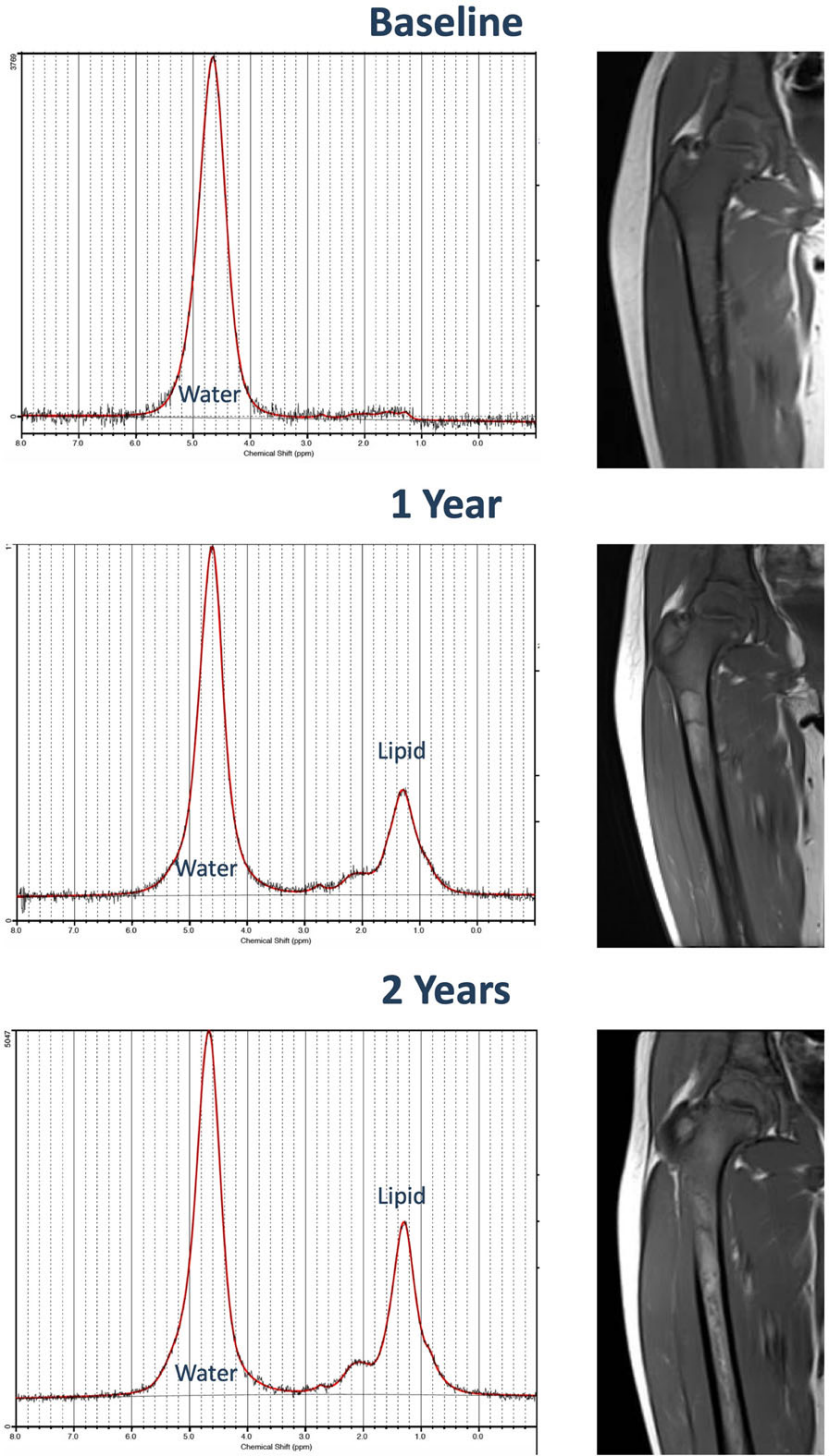
Magnetic resonance spectroscopy assessment of marrow response to treatment. Gaucher disease patient with first imaging at 15 years of age subsequently treated with ERT. Magnetic resonance spectroscopy spectra (left) show increasing fat-fraction on follow-up examinations. Conventional T1-weighted coronal images (right) of the right femur demonstrate diffuse marrow infiltration with subtle fat infiltration with time

Currently, quantitative MRI-based fat fraction measurements have been applied to objectively assess marrow involvement in Gaucher disease. Quantitative chemical-shift imaging (QCSI) using Dixon-based techniques and MRS are both capable of reliably measuring bone marrow fat fraction [85–90]. Work by Maas and colleagues has suggested a cut-off of 0.23 for a fat fraction ratio—values lower than this suggest a markedly elevated risk of bone-related complications [91]. Early studies also identified a correlation between marrow fat fractions and spleen size, suggesting an association between bone marrow and visceral disease severity [84]. One small MRS study noted significantly lower fat fractions in the proximal femurs but not the lumbar spine of mostly asymptomatic children with Gaucher children compared with age-matched controls [92]. Treatment-related responses in bone marrow fat fractions have been demonstrated in response to ERT (Fig. 9) [93–95]. A fat fraction of 0.37 (derived from normal controls) or more is proposed as a reference goal for treatment response [72]. Marrow fat quantification may serve as a means of prognostication and identifying nonresponders with responses noted as early as 1 year [91, 93, 95].

The importance of understanding developmental increases in marrow fat over the lifespan beginning first in the peripheral skeleton as well as normal variation in bone marrow fat fractions cannot be understated. Patient age, sex, and body mass index should be considered in the interpretation of these values, ideally in the context of normative data relevant to the measurement method [88, 96–98]. Importantly, untreated Gaucher patients do not seem to exhibit normal expected age-related increases in fat fractions [93]. Adoption of QCSI for fat fraction measurements has been stymied for decades outside a few selected academic medical centers due to technical requirements, but the recent release of commercially available sequences may lead to a revival of fat fraction quantification [88, 99].

DWI may also hold promise with some evidence of decreased vertebral marrow ADC reflecting increased cellularity corresponding with disease status [100]. Last, iron filtration in marrow has been quantified in Gaucher disease with elevated R2* values in femoral and vertebral marrow compared to matched healthy controls [48].

##### Metabolic imaging assessment

Nuclear medicine studies are not routinely used for routine follow-up assessment of Gaucher disease at most institutions, although 99 m-Tc-Sestamibi scintigraphy has been studied as an alternative means of quantifying bone marrow involvement with good agreement with MRI-based semi-quantitative scoring [101, 102]. Treatment-related changes have been successfully observed using 99 m-Tc-Sestamibi scintigraphy [103]. Other scintigraphy methods are largely ineffective at quantifying marrow involvement as typical bone radiotracers are only taken up by normal marrow, but 99 m-Tc-MDP could potentially be helpful in discerning between bone crises and osteomyelitis, although this observation is not well validated [22]. Nuclear medicine-based techniques are hampered by limited spatial resolution and inherent ionizing radiation exposure. While sestamibi patterns do not appear to be affected by age-related marrow conversion, ionizing radiation exposure incumbent in this technique precludes adoption for pediatric imaging [32]. In the future, targeted assessment of disease involvement may be possible with promising experimental positron emission tomography data using a 18F-labeled glucocerebrosidase [104]. In current practice, superior assessment of both marrow, osseous complications, and soft tissues has made MRI the modality of choice for the evaluation of musculoskeletal manifestations of Gaucher disease.

#### Osteopenia

One of the earliest skeletal manifestations of Gaucher disease type 1 in children is osteopenia [31]. The development of osteopenia in Gaucher disease is multifactorial with abnormal osteoclast function and decrease in bone formation as possible etiologies reviewed in detail elsewhere [105]. Abnormal trabecular bone density has been observed in the absence of focal abnormalities or remodeling and cortical thinning is noted with marrow expansion on CT [81, 82]. Decreased bone density is appreciated qualitatively on radiographic examinations and quantitatively assessed using dual-energy X-ray absorptiometry (DXA) assessment of bone mineral density (BMD). Z-scores from multiple sites are lower for Gaucher disease patients compared with healthy individuals [106]. Bone density has also been observed to increase in response to treatment [28, 107, 108]. Technical difficulties may be encountered in interpreting BMD in younger children, patients with compression fractures, and bone infarction [61]. Nonetheless, BMD retains an important role in predicting the risk of osteonecrosis and pathologic fracture in adult patients [109].

#### Osseous complications

Progressive osseous involvement in type 1 Gaucher disease may begin with painful bone crises that may eventually result in osteonecrosis (also termed avascular necrosis and bone infarction) in about half of patients that may be attributed to packing of the marrow with Gaucher cells leading to thrombosis in situ within the marrow [57, 110]. Osteonecrosis most commonly involves the femoral head and neck, proximal humeri, tibias, and vertebrae [22]. Upper extremity involvement seems to occur in patients with greater global bone marrow involvement [78]. Incumbent pathologic fractures occur in about one-tenth to one-quarter of patients and often require specialized orthopedic management including arthroplasty detailed elsewhere [110–113]. The frequency of osseous complications is thought to mirror that of other organ involvement severity; one study showed a significant difference in liver volumes between patients with and without osteonecrosis [56]. Prior to the advent of ERT, thoracic kyphosis and vertebral body collapse following severe osteopenia were commonly noted complications in affected children with resultant morbidity and deformity [114]. Bone crises are now infrequently encountered in pediatric patients in the era of ERT [10]. Osteomyelitis is a relatively infrequent complication noted in 6% of patients in one cohort [110].

Radiographic detection of early osseous complications such as osteonecrosis and bone infarct may be difficult, often being occult on initial imaging with subsequent development periosteal reaction that may be confused with osteomyelitis (“pseudo-osteomyelitis”) [22, 67]. Focal accumulation can result in lytic lesions visible radiographically [32]. Later stages of osteonecrosis demonstrate serpentine sclerosis radiographically. Radiographs are helpful, however, in the initial workup of acute pain to assess for pathologic fracture [115]. Late sequelae including collapse of the femoral heads, proximal humeri, and vertebral bodies can also be appreciated on radiographs along with early onset osteoarthritis [10].

**Fig. 10:**
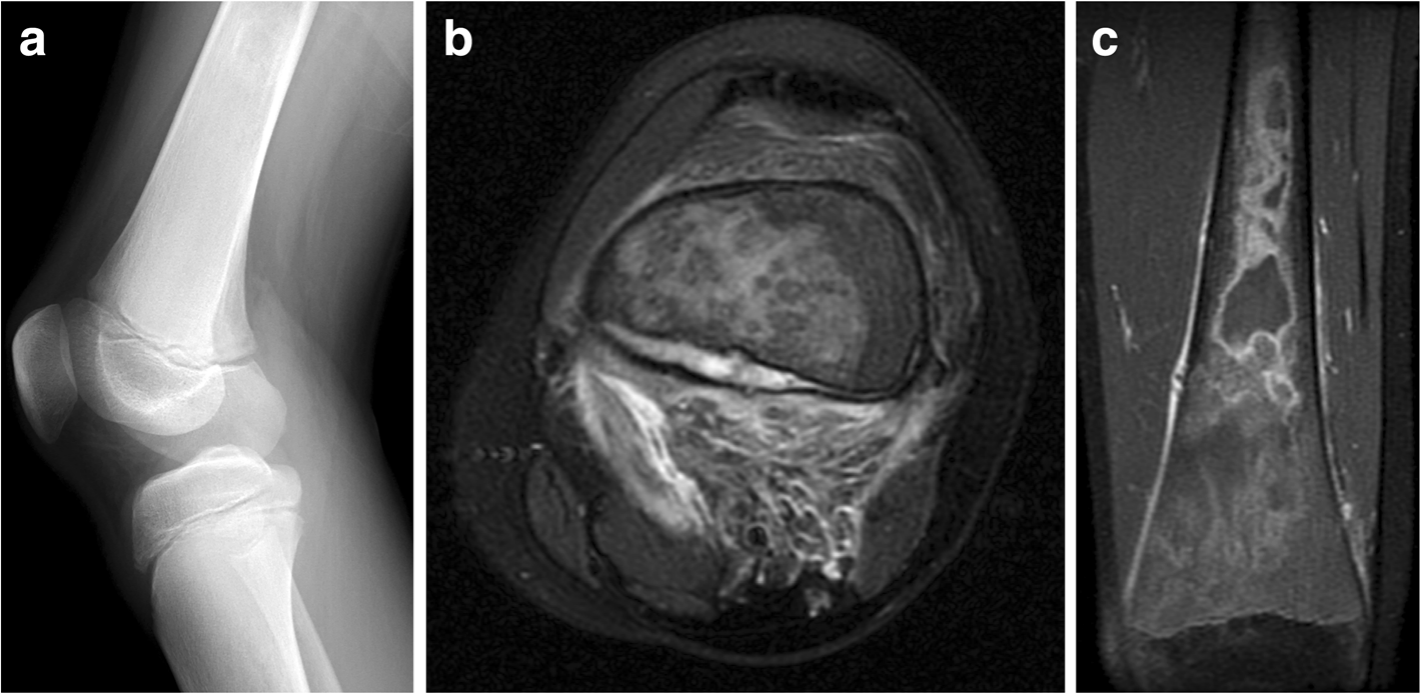
Bone infarct and subperiosteal hemorrhage mimicking osteomyelitis in Gaucher disease. A 13-year-old male presented with atraumatic knee pain and swelling. Initial lateral knee radiograph (**a**) showed subtle periosteal reaction along the posterior metadiaphysis of the distal femur and ill-defined intramedullary sclerosis within the femoral shaft. Follow-up MRI demonstrated intramedullary marrow edema and focal subperiosteal fluid with surrounding inflammatory changes in the popliteal fossa thought to be related to subperiosteal hemorrhage on axial T2-weighted image (**b**). Coronal post-contrast T1 fat-saturated image (**c**) demonstrated peripheral serpentine enhancement of areas of bone infarction within the distal femur. The patient was subsequently diagnosed with Gaucher disease after initial workup for hematopoietic malignancy

Given these limitations, assessment of acute osseous complications is best performed with initial radiographs followed by prompt MRI assessment [72]. Bone crises may be seen with focal marrow edema on fluid-sensitive sequences [10]. With progressive ischemic insults, osteonecrosis can develop and is evident as T1-weighted hypointense signal with heterogeneous T2-weighted hyperintense signal often with a “double line” of low and high signal reflecting bone infarction [67]. This focal bone involvement can progress further with collapse of the femoral heads and vertebral bodies encountered in severe marrow disease [10]. Other associated complications can be seen including cortical disruption and subperiosteal hemorrhage (Fig. 10) in the setting of massive medullary expansion and cortical thinning, which may also mimic osteomyelitis [116, 117].

Bone-related malignancies may be slightly more common in Gaucher disease, perhaps related to osteonecrosis as a predisposing factor. MRI may often be warranted for evaluation of discrete lytic lesions given the incidence of malignant bone lesions such as multiple myeloma, osseous lymphoma, sarcomas, and malignant epithelioid hemangioendothelioma [118–120]. Particular attention should be paid to enlarging lesions and those with morphology dissimilar to more common Gaucher disease-related osteopenia, marrow infiltration, and osteonecrosis.

### Cardiopulmonary involvement

#### Pulmonary infiltration

As with the liver, spleen, and bone marrow, Gaucher cells can infiltrate in the lungs, typically depositing within interstitial spaces. Such lung involvement is quite rare, more common in the neuronopathic type, nd not likely to occur as an initial manifestation of this disease [10]. Reticulonodular opacities may be seen on chest radiography, but chest CT evaluation is more useful in depicting centrilobular nodules, ground glass opacities, interstitial, and bronchial wall thickening [22].

#### Pulmonary hypertension

There are multiple proposed etiologies for pulmonary hypertension in Gaucher patients. Pulmonary hypertension is attributable to perivascular infiltration of Gaucher cells or secondary to hepatic disease [22]. An early report of echocardiography in 134 adult Gaucher disease patients identified pulmonary hypertension in approximately one in ten treated patients and none in untreated patients, raising concern for possible treatment-related pulmonary hypertension [121]; however, a subsequent follow-up of children screened with echocardiography did not find pulmonary hypertension in this younger patient population [122]. Therefore, routine screening of pediatric Gaucher disease patients for pulmonary hypertension is not indicated.

#### Cardiac involvement

The extent of cardiac involvement in Gaucher disease is uncertain with small numbers of mitral valve prolapse and valvular calcifications reported in a few patients with homozygous D409H mutations [123]. Given the infrequent occurrence of cardiac involvement, most authors only recommend baseline echocardiographic screening in children with Gaucher disease to exclude abnormalities with others also recommending follow-up echocardiography in children with homozygous D409H mutations [122, 123]. A more recent investigation of cardiac MRI in a small number of Gaucher disease patients noted left atrial enlargement in three of nine patients [124], and others have implied an association between Gaucher disease and left ventricular diastolic dysfunction on echocardiography, suggesting myocardial infiltration as a possible etiology [125]. Further systematic investigation is needed to assess the frequency of cardiac involvement in Gaucher disease, and the possible relevance of imaging for screening for subclinical disease.

## Recommended imaging protocols

There is a clear need for an objective and reproducible scoring system capable of assessing disease severity to standardize patient monitoring. While clinical scoring methods (PGS3 and GD-DS3) are useful tools for estimating disease severity, these scoring methods do not assess diffuse hepatic disease such as fibrosis, iron accumulation, fat infiltration, and portal hypertension, nor do they assess the full extent of asymptomatic marrow involvement with inclusion of only subjective imaging assessment of marrow involvement.

While ultrasound is affordable and may be sufficient for the assessment of organ volumes and liver lesion screening, there is increasing use of MRI in Gaucher disease with most authorities considering MRI the standard of care [24, 45]. As highlighted throughout this article, there are additional findings on newer imaging methods worth assessing in Gaucher disease patients that cannot be imaged using conventional MRI, ultrasound, and/or radiography. For this reason, we advocate the routine use of an MRI protocol that provides both qualitative and quantitative information of multiple organ systems to provide a comprehensive assessment of disease involvement in Gaucher disease. In younger children under the age of 6 years, sedation may be needed to perform MRI; abbreviated protocols, child life, and distraction techniques may reduce the need for sedation in children [23]. In institutions where particular resources are constrained or unavailable, suitable alternative imaging techniques may be employed acknowledging potential limitations summarized in Table 4. Some examples of alternatives to MRI-based modalities include carefully acquired ultrasound measurements of organ volumes using standardized methods by selected readers and ultrasound elastography methods. However, reliable determination of marrow infiltration requires MRI or nuclear medicine studies.

### Liver and spleen MRI

Diagnostic imaging of hepatic and splenic involvement should consist of sequences tailored for volumetric organ measurement and screening for focal lesions. In conjunction with these conventional acquisitions, MR elastography should be considered, when available, to provide non-invasive assessment of liver and splenic stiffness. While the role of elastography in the clinical management of hepatic fibrosis in Gaucher disease is not yet established, acquisition of organ stiffness measurements may afford insights into disease and treatment effects in the future with wider clinical adoption. It is important to note that elastography results should be interpreted in the context of known limitations including inaccuracy of gradient recalled echo(GRE)-based elastography in patients with iron deposition and massive ascites [42]; as such, performance of both GRE and spin-echo echo-planar imaging elastography sequences is recommended. In addition, assessment of fat fractions and iron quantification may be helpful in providing additional quantitative information for disease severity and treatment response assessment, although these methods also require additional validation in larger populations.

Newer proton density fat fraction (PDFF) sequences allow for single breath-hold acquisition of the entire liver to provide triglyceride fat fractions, R2* mapping, and some also provide automated liver volumes (mDIXON Quant, Philips Healthcare; LiverLab, Siemens Healthineers; IDEAL-IQ, GE Healthcare) [126–128]. These measurements are reliable and highly reproducible across vendors, making them ideal for multi-center investigations that are essential in studying rare conditions such as Gaucher disease [127, 128]. However, these measurement packages often require significant capital and are not yet widely available outside of major research institutions.

Single-voxel MRS for fat fraction measurement may also be considered as a supplement or alternative if PDFF sequences are not available. DWI is included in our protocol as it may also be provide indirect assessment of organ cellularity with ADC as a possible quantitative marker of disease severity [37].

If suspicious focal lesions are noted on this surveillance evaluation, dedicated lesion work-up with dynamic hepatocyte-specific contrast-enhanced MRI, multiphase contrast-enhanced CT, or contrast-enhanced ultrasound should be recommended, depending on institutional resources [54, 129, 130].

### Bone marrow MRI

Qualitative imaging of both the axial (lumbar spine) and appendicular skeleton (proximal femurs) is important for assessment of global marrow involvement, as these sites may respond differently to treatment [64]. While a few recent studies have explored the use of whole-body MRI to screen the entire skeleton, none of the cases of humeral involvement were asymptomatic and therefore screening of the upper extremities may be less salient [78]. As emphasized throughout this article, quantitative measurement of bone marrow fat fractions is of increasing importance in estimating disease severity and tracking treatment response. Similar PDFF acquisitions (mDIXON Quant, Philips Healthcare; LiverLab, Siemens Healthineers; IDEAL-IQ, GE Healthcare) employed to measure fat fractions in the liver may be employed to measure fat fractions in the proximal femur (neck) and lumbar spine (L5), although marrow measurements using these sequences have not been validated in Gaucher disease [88]. Single-voxel MRS is a reliable alternative quantitative method that has been used in Gaucher disease and shows good concordance with Dixon-based methods [92, 131], although acquisition times may be greater for this method. MRS additionally requires expertise in analysis for fat fraction determination, but may be more reliable for lower fat fractions. Both PDFF and MRS generally require the contributions of a medical physicist dedicated to the successful implementation of these protocols. Therefore, institutions without such expertise in quantitative imaging may rely on conventional acquisitions and subjective assessment of marrow involvement by radiologists familiar with semi-quantitative scoring methods such as BMB.

### Frequency of comprehensive MRI evaluation

Discrete parameters for the frequency of follow-up of Gaucher disease patients with imaging are informed by consensus recommendations and institution-specific practices [18, 24, 132, 133]. Comprehensive imaging evaluation of the liver, spleen, and bone marrow of the femurs and lumbar spine should be performed at initial diagnosis, annually for asymptomatic, untreated pediatric patients and biannually for asymptomatic, untreated adults. While some recommend less frequent skeletal imaging evaluation of asymptomatic patients, subclinical marrow involvement appears to be relatively common and asymptomatic individuals may benefit from regular monitoring [24, 58]. Abdominal imaging is generally recommended on a more frequent basis, every 6 months, in younger pediatric patients [18]. Given the convenience of the previously described comprehensive MRI evaluation (when appropriate sequences and equipment are available) and the observation that musculoskeletal complications indicative of treatment failure sometimes only brought to clinical attention by serial imaging [110], we recommend annual MRI of the bone marrow and abdominal organs. Follow-up evaluation in actively treated patients is typically performed annually as well, although marrow responses may plateau after 4 to 5 years of successful therapy [24, 93]. Ultimately, the frequency and nature of imaging follow-up may need to be adjusted on an individual basis dependent on the availability of imaging techniques, severity of involvement, and trends on prior imaging assessments.

### Bone density assessment

As part of clinical severity scoring and recommended care for patients, bone mineral density measurement is recommended beginning at 6 years of age with follow-up obtained every 1 to 2 years [14, 18, 31]. DXA in children should be performed at institutions with technical and interpretive expertise specific to pediatric populations [18, 23]. DXA may be performed less frequently in adults depending on extent of marrow infiltration and risk of osteoporosis-related complications.

### Assessment of bone complications

Regional radiographic assessment of acute bone pain should be the first-line examination to assess for the presence of pathologic fracture [24]. However, we advocate a low threshold for the evaluation of affected osseous structures with MRI, given the markedly improved sensitivity for osteonecrosis and collapse [72].

### Other imaging assessments

Decisions regarding routine imaging assessment of cardiac abnormalities with echocardiography and cardiac MRI are beyond the scope of this review with some authors recommending baseline echocardiographic screening in all ages at diagnosis and follow-up evaluation in adults on treatment [121, 122]. These clinical approaches to cardiac involvement may be best individually tailored on the basis of clinical presentation and genotype in the absence of more definitive evidence to support widespread use. Chest CT is valuable in the assessment of patients with suspected primary pulmonary involvement or secondary hepatopulmonary syndrome and may be helpful in symptomatic patients, but routine use of chest CT is not recommended due to the rarity of these abnormalities in asymptomatic type 1 patients [10].

## Conclusions

Conventional imaging of non-neuronopathic Gaucher disease underestimates multisystem organ involvement in Gaucher disease, and recent clinical and experimental evidence shows a significant role of multifaceted processes of inflammation, chemotaxis, iron deposition, and fibrosis, even in treated patients. Quantitative bone marrow fat fraction measurements have been touted as a reliable marker of skeletal involvement for treatment response assessment but have been relegated to selected academic medical centers previously. Newer commercially available quantitative MRI methods enable efficient quantification of liver, spleen, and bone marrow fat fraction values and a comprehensive imaging protocol is introduced in this article to take advantage of this opportunity to better characterize this complex disease and guide personalized therapy.

## Abbreviations

ADC: Apparent diffusion coefficient
BMB: Bone marrow burden
BMD: Bone mineral density
CCL18: Chemokine ligand 18
DWI: Diffusion-weighted imaging
ERT: Enzyme replacement therapy
GBA: Glucocerebrosidase
GD-DS3: Gaucher Disease Type 1 Severity Scoring System
HCC: Hepatocellular carcinoma
MRE: Magnetic resonance elastography
MRS: Magnetic resonance spectroscopy
PDFF: Proton density fat-fraction
PGS3: Pediatric Gaucher Severity Scoring System
QCSI: Quantitative chemical shift imaging
S-MRI: Spanish MRI
SRT: Substrate reduction therapy
SWE: Shear wave elastography
TE: Transient elastography
